# Supplementary material for: Turn air-captured CO2 with methanol into amino acid and pyruvate in an ATP/NAD(P)H-free chemoenzymatic system
Source: Nat Commun. 2023 May 15;14:2772. doi: 10.1038/s41467-023-38490-w (PMC10185560; doi:10.1038/s41467-023-38490-w)
Supplement: Supplementary file 1 — Supplementary Information [file 41467_2023_38490_MOESM1_ESM.pdf]

Supplementary information

Turn air-captured CO_2 with methanol into amino acid and pyruvate in an ATP/NAD(P)H-free chemoenzymatic system

Jianming Liu[1#], Han Zhang[1#], Yingying Xu[2], Hao Meng[2], An-Ping Zeng[1*]

[1]Center of Synthetic Biology and Integrated Bioengineering, School of Engineering, Westlake University. 600 Dunyu Road, Xihu District, Hangzhou 310024, Zhejiang Province, China

[2]Beijing Advanced Innovation Center for Soft Matter Science and Engineering, Beijing University of Chemical Technology, Beijing, China

[#]Contributed equally to this work

*** Corresponding author: Prof. An-Ping Zeng**

zenganping@westlake.edu.cn

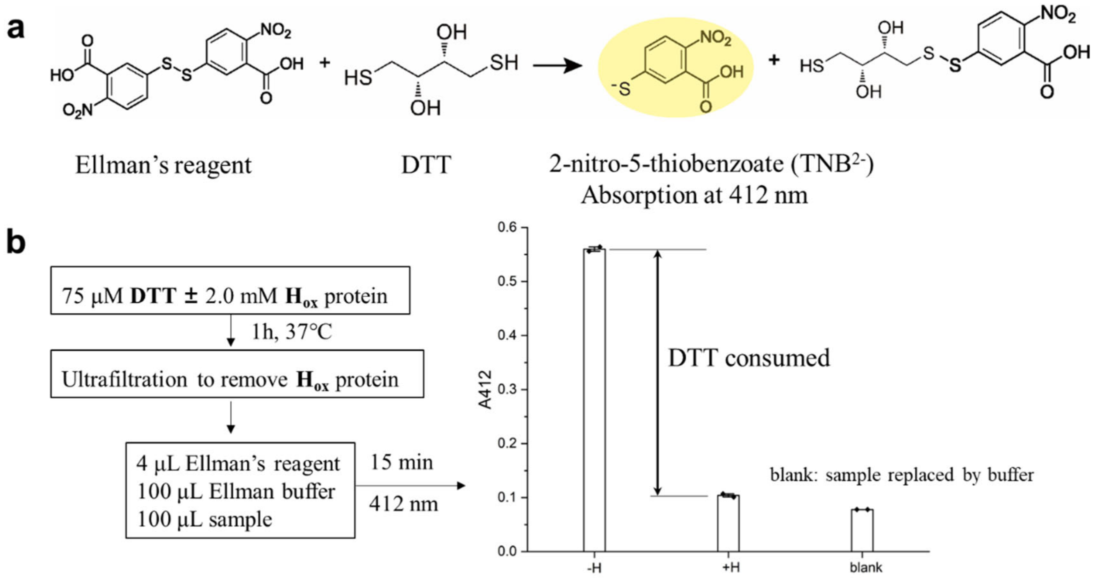

Supplementary Fig. 1 Measurement of DTT consumption in the process of electron-transfer to H_{ox} using Ellman's reagent. **a**, the chemical reaction between DTT and Ellman's reagent can generate a yellow compound 2-nitro-5-thiobenzoate (TNB^{2-}) that has an absorption at 412 nm. **b**, Electron transfer from DTT to H_{ox}. In the assay, a low concentration of DTT of 75 µM and 2.0 mM H_{ox} were mixed together at 37 °C for 1 h, after that, the mixture was separated using ultrafiltration. The remaining DTT was measured. Bars represent the mean change ± SEM of 2 independent experiments.

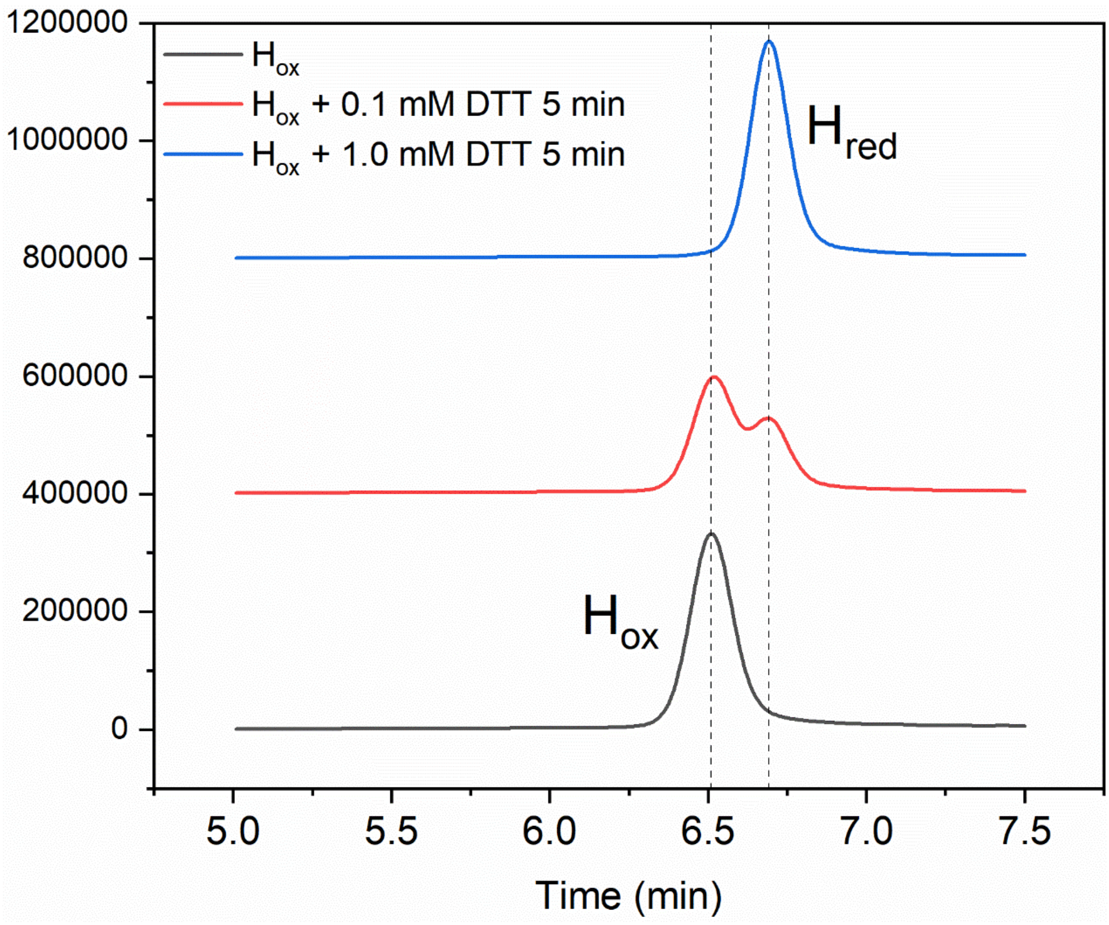

Supplementary Fig. 2 Quantification of the conversion of H_{ox} to H_{red} in the presence of DTT using HPLC. H_{ox} was fresh H protein purified from the recombinant *E. coli* BL21 (pET28a-H) cells. To make H_{red}, 50 μM H_{ox} and 0.1 mM or 1.0 mM DTT were reacted for 5 mins. In the presence of 1.0 mM DTT, 50 μM H_{ox} was completely reduced to H_{red} in 5 mins and maintained stable in such an environmental condition (2-h and 10-h samples displayed the same in chromatographs).

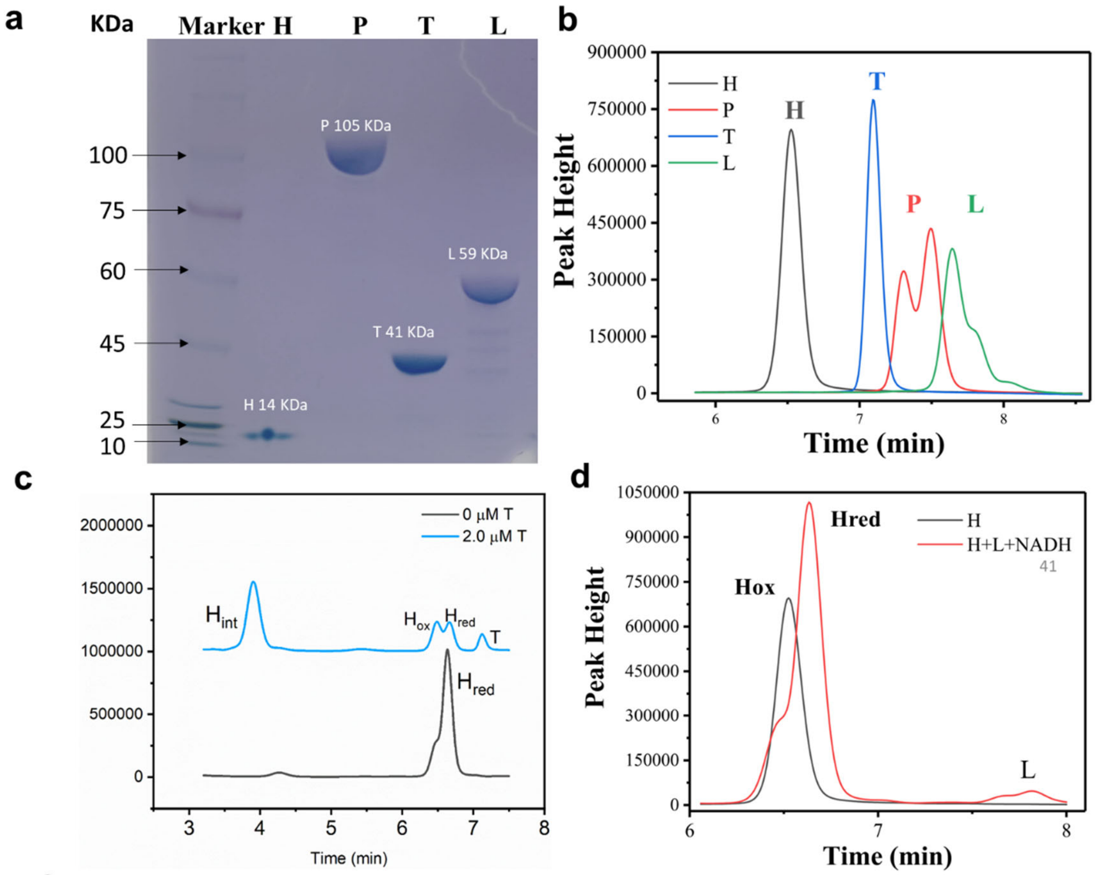

e

$$H_{red} \xrightarrow{T} H_{int}$$

$$H_{ox} \xrightarrow[DTT]{L} H_{red}$$

$$H_{ox} \xrightarrow{GCS} \text{Glycine}$$

f

T	~373 mmol H_{int}/μmol T/min
L	~0.67 mmol H_{red}/ μmol L/min
DTT	~10 mmol H_{red}/mmol DTT/min
GCS	~0.03 mmol Glycine/ μmol P/min

Supplementary Fig. 3 Quantitative analysis of the rGCS components and reactions. **a**, SDS-PAGE analysis for purified H, P, T and L proteins (One representative figure is shown from at least 3 independent experiments). **b**, The rGCS proteins were analyzed based on the HPLC method using a Shimadzu LC-2030C system with a ZORBAX 300SB-C18 column (4.6×250 mm, 5 μM) and monitored at 210 nm using a DAD-3000 diode array detection. **c**, Measurement of the reaction catalyzed by T protein in the absence of DTT. We prepared H_{red} from 100 μM H_{ox} using 5 μM L protein and 20 mM NADH for 0.5 h. Then 2 μM T protein, 5 mM NH_4HCO_3, 2 mM HCHO and 0.5 mM THF were added to the system. The formation of H_{int} was measured after 10 mins, which was used to calculate the T reaction rate. **d**, H_{ox} to H_{red} with L and NADH. The assay contains 100 μM H_{ox}, 5 μM L protein and 20 mM NADH. **e**, The reactions used to calculate the velocity. **f**, The approximate velocity for each reaction.

a

	DTT1	DTT2	L1	L2
DTT	20 mM	5 μM		
L			5 μM	1 mM
NADH			10 mM	
HCHO	10 mM			
THF	0.5 mM			
NH_4HCO_3	50 mM			
PLP	25 μM			
H	10 μM			
T	5 μM			
P	5 μM			

b

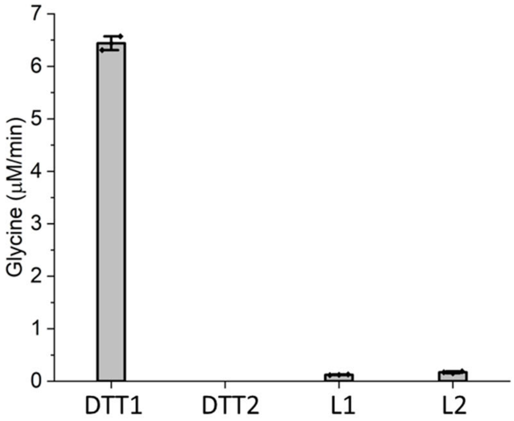

Supplementary Fig. 4 Glycine production in the presence of DTT or L protein at comparable concentrations. **a**, The components and their concentrations used in the reaction. **b**, Glycine production rate under different conditions. Bars represent the mean change ± SEM of 3 independent experiments.

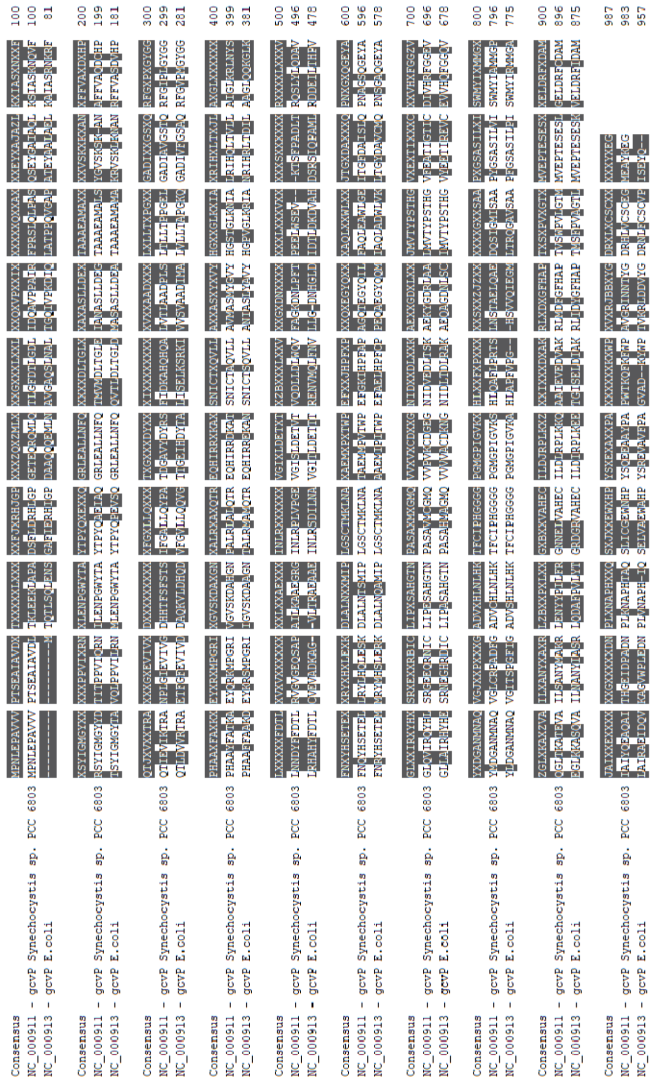

Supplementary Fig. 5 P protein (NCBI No. WP_010873139.1) from *Synechocystis* sp. PCC 6803 and P protein (NCBI No. WP_112929453.1) from *Escherichia coli* K-12 MG1655 have a 57.2% similarity in amino acids.

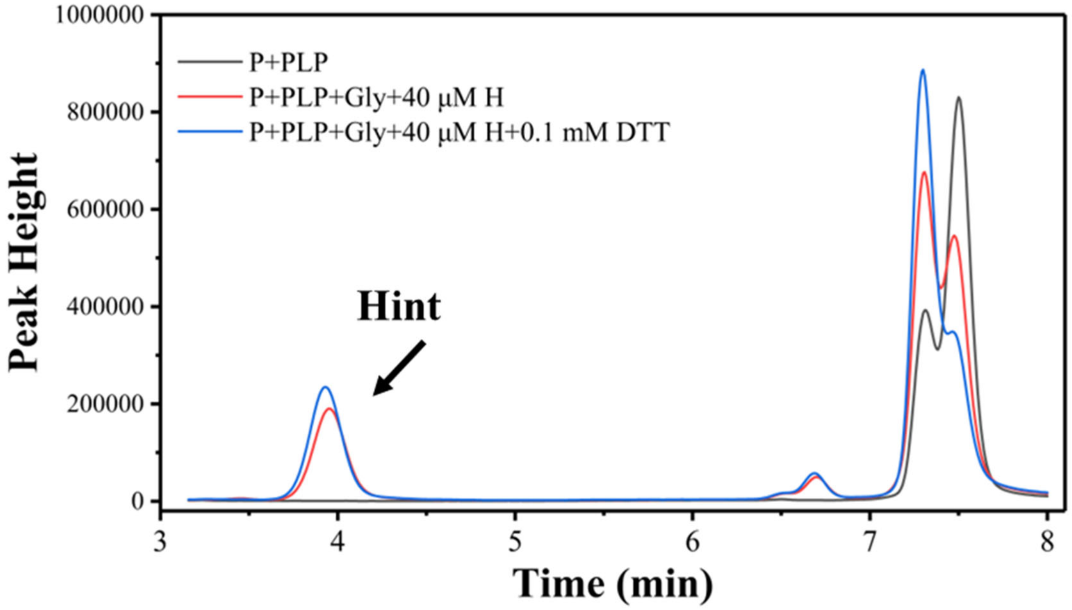

Supplementary Fig. 6 DTT accelerated the conversion from the oxidized P state to its reduced state, resulting in an increased production of H_{int} from H_{ox} in the glycine cleavage reaction using glycine as the substrate.

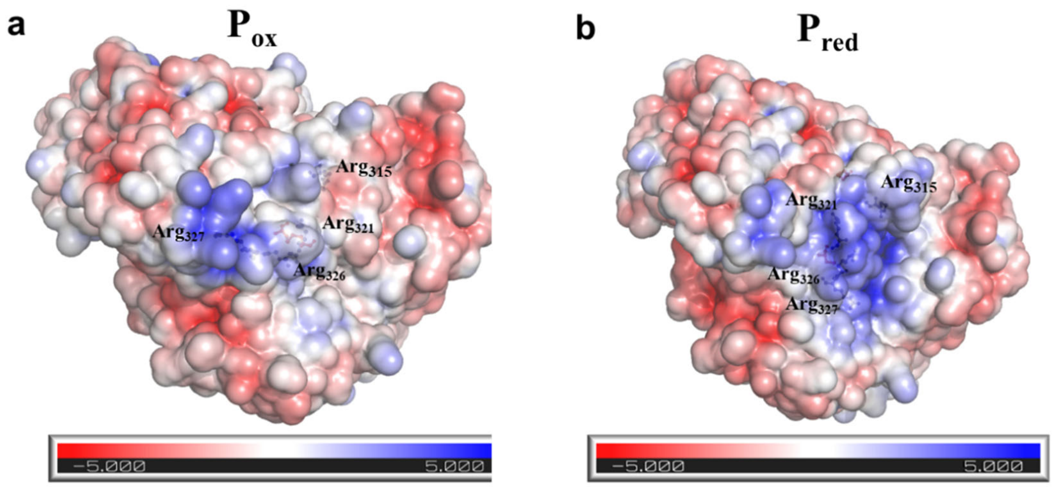

Supplementary Fig. 7 Electrostatic surface analysis of the oxidized state (**a**) and reduced state (**b**) of P protein. Electrostatic surfaces are colored blue in the positive region and red in the negative region.

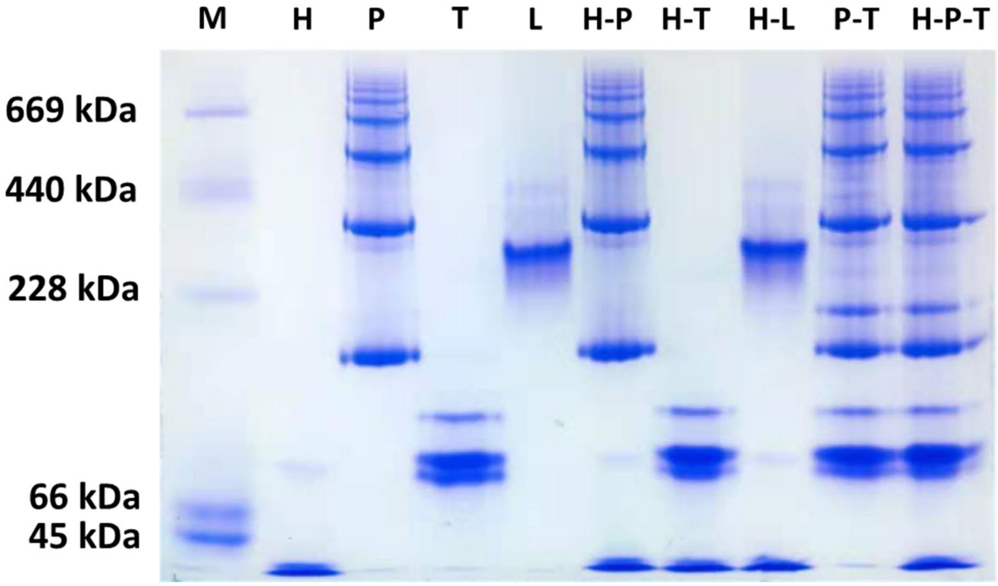

Supplementary Fig. 8 Native-PAGE of the rGCS proteins and their mixtures. M, Protein marker; H, 30 μM H protein; P, 4.3 μM P protein; T, 10.8 μM T protein; L, 8.7 μM L protein; H-P, 30 μM H protein + 4.3 μM P protein; H-T, 30 μM H protein + 10.8 μM T protein; H-L, 30 μM H protein + 8.7 μM L protein; P-T, 4.3 μM P protein +10.8 μM T protein; H-P-T, 30 μM H protein + 4.3 μM P protein + 10.8 μM T protein (One representative figure is shown from 3 independent experiments).

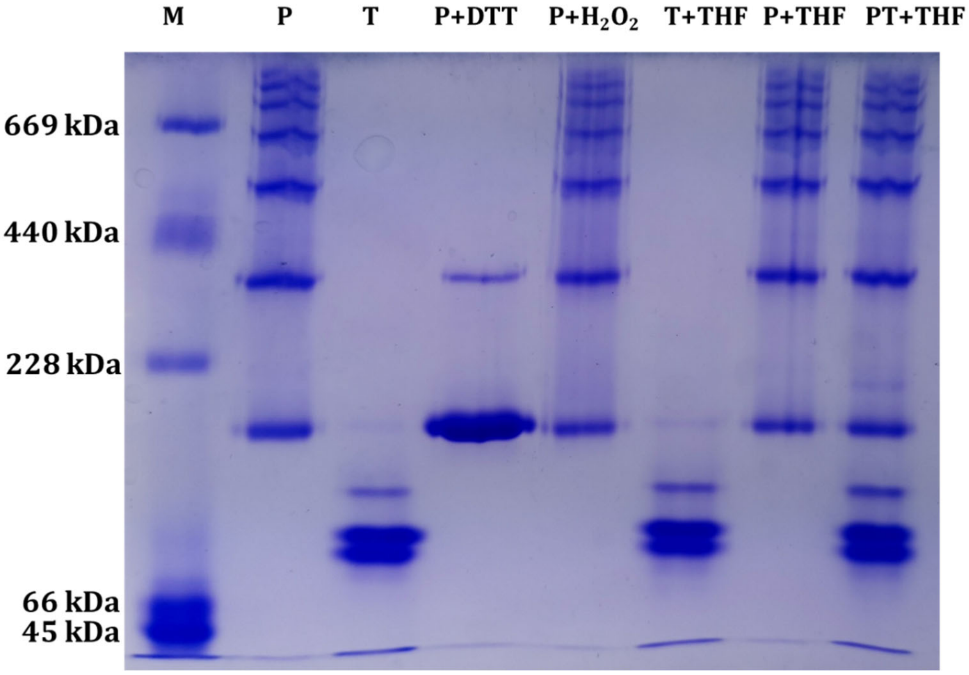

Supplementary Fig. 9 Native-PAGE of P, T proteins with DTT, H_2O_2 and THF. M, Protein marker;P, 4.3 μM P protein; T, 10.8 μM T protein; P+DTT, 4.3 μM P protein + 50 mM DTT; P+H_2O_2, 4.3 μM P protein + 50 mM H_2O_2; T+THF, 10.8 μM T protein + 1 mM THF; P+THF, 4.3 μM P protein + 1 mM THF; PT+THF, 4.3 μM P protein +10.8 μM T protein + 1 mM THF (One representative figure is shown from 3 independent experiments).

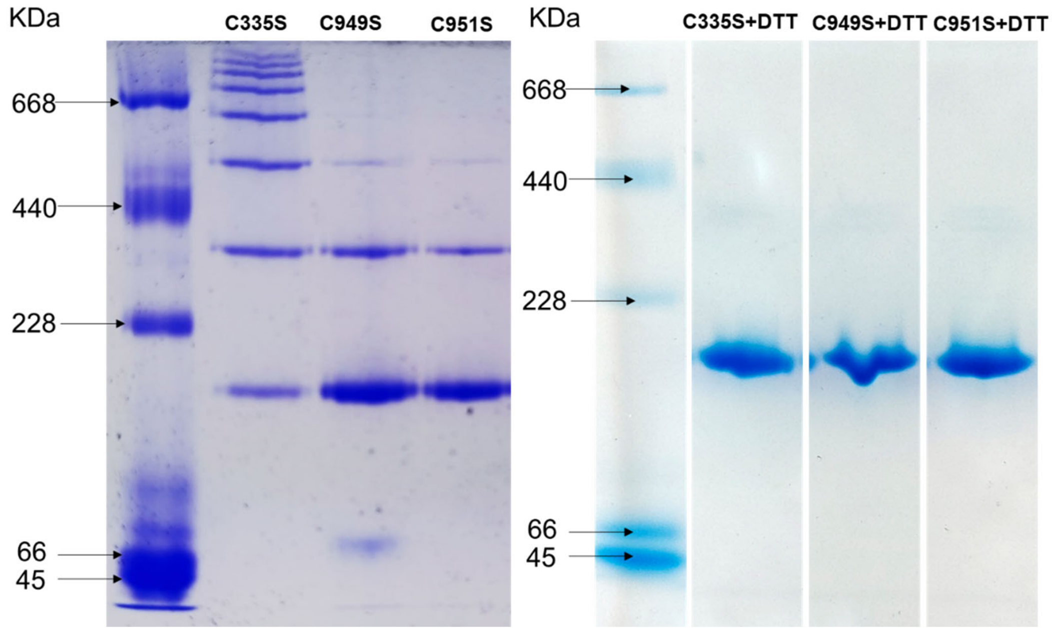

Supplementary Fig. 10 Native-PAGE of C335S, C949S, C951S proteins with DTT. C335S, 4.3 µM; C949S, 4.3 µM; C951S, 4.3 µM. C335S+DTT, C335S protein + 50 mM DTT; C949S+DTT, 4.3 µM C949S protein + 50 mM DTT; C951S+DTT, 4.3 µM C951S protein + 50 mM DTT (One representative figure is shown from 3 independent experiments).

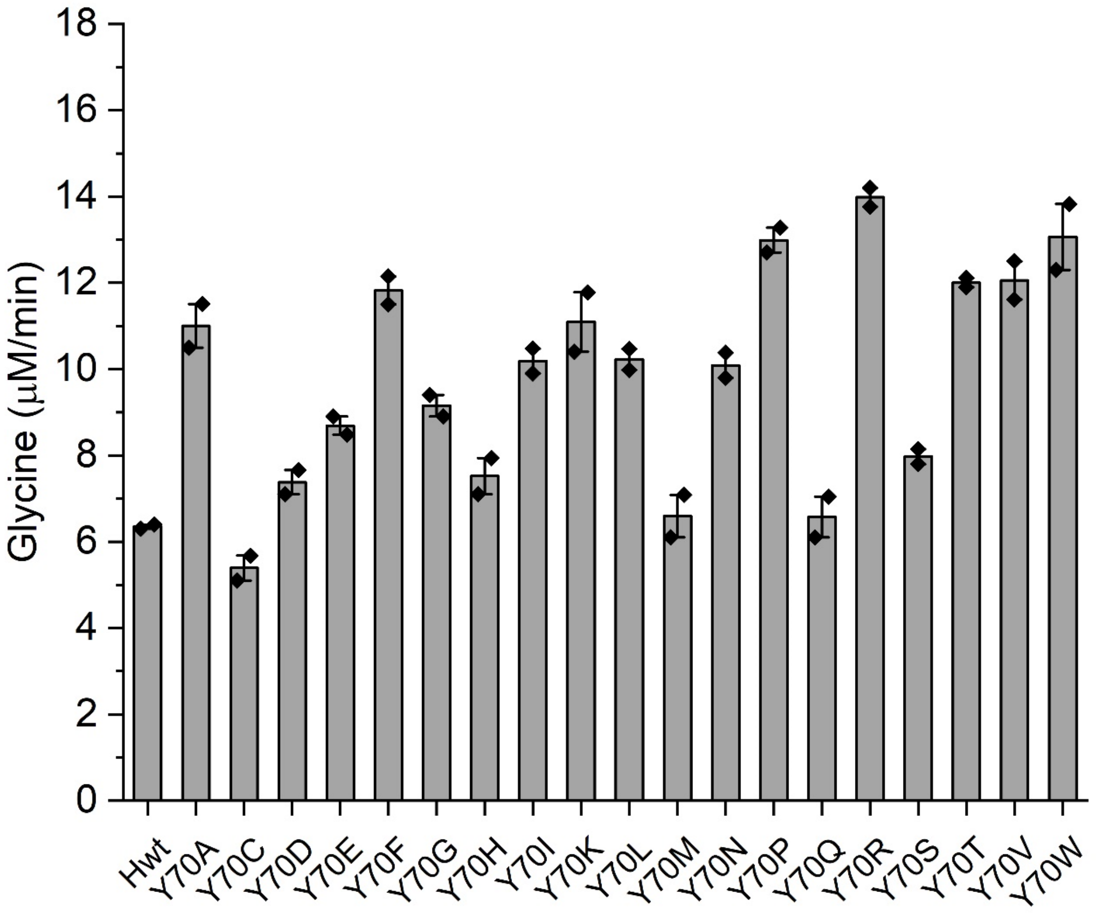

Supplementary Fig. 11 Glycine formation rate using a Y70-site saturated mutagenesis library. Bars represent the mean change ± SEM of 2 independent experiments.

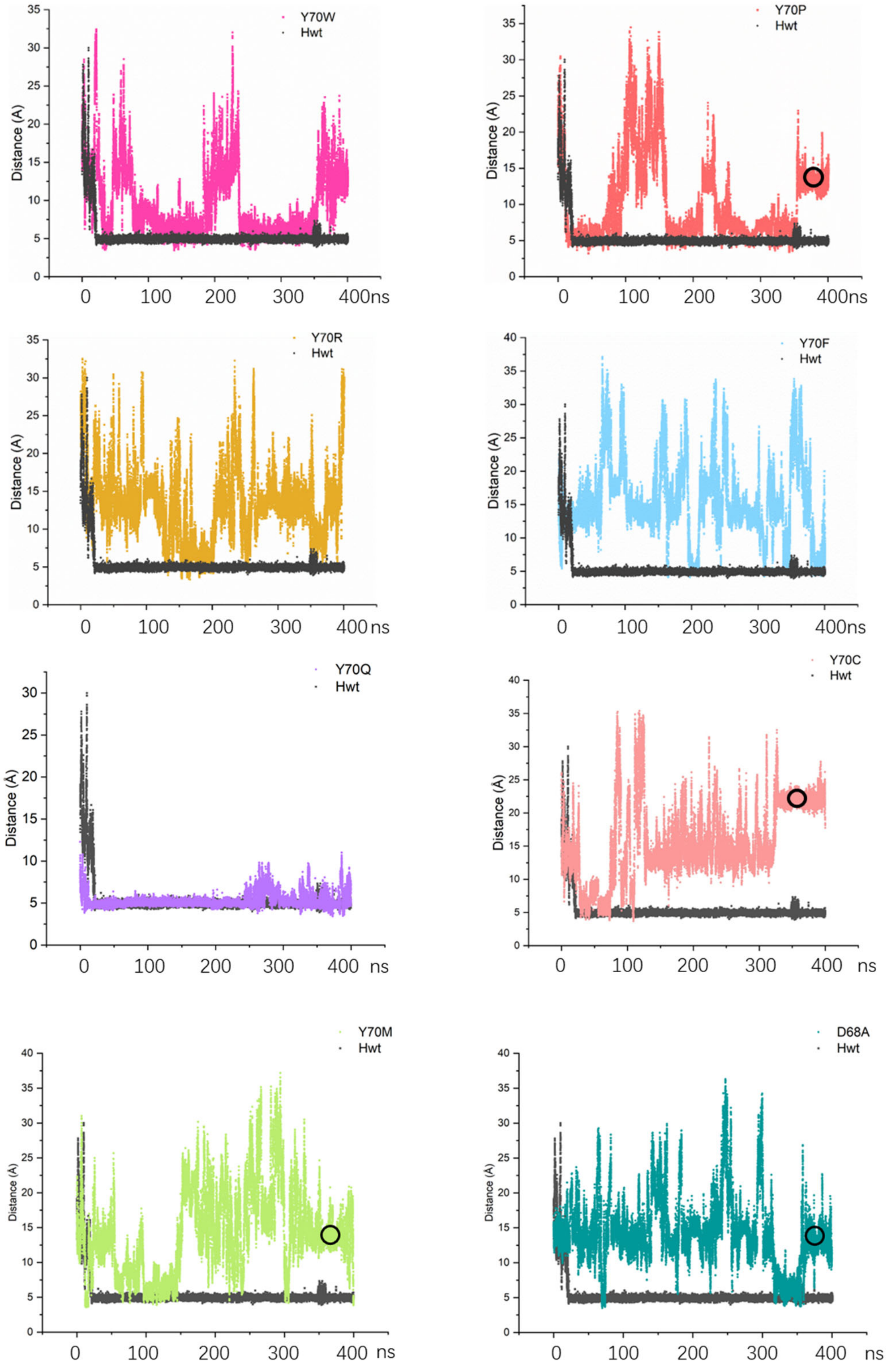

Supplementary Fig. 12 Molecular dynamics simulations of H_{wt} and Y70W, Y70P, Y70R, Y70F, Y70Q, Y70C, Y70M and D68A. The distance between Cα at E12 of the H protein and the N atom located in the aminomethyl moiety is measured for the first 400 ns in simulations.

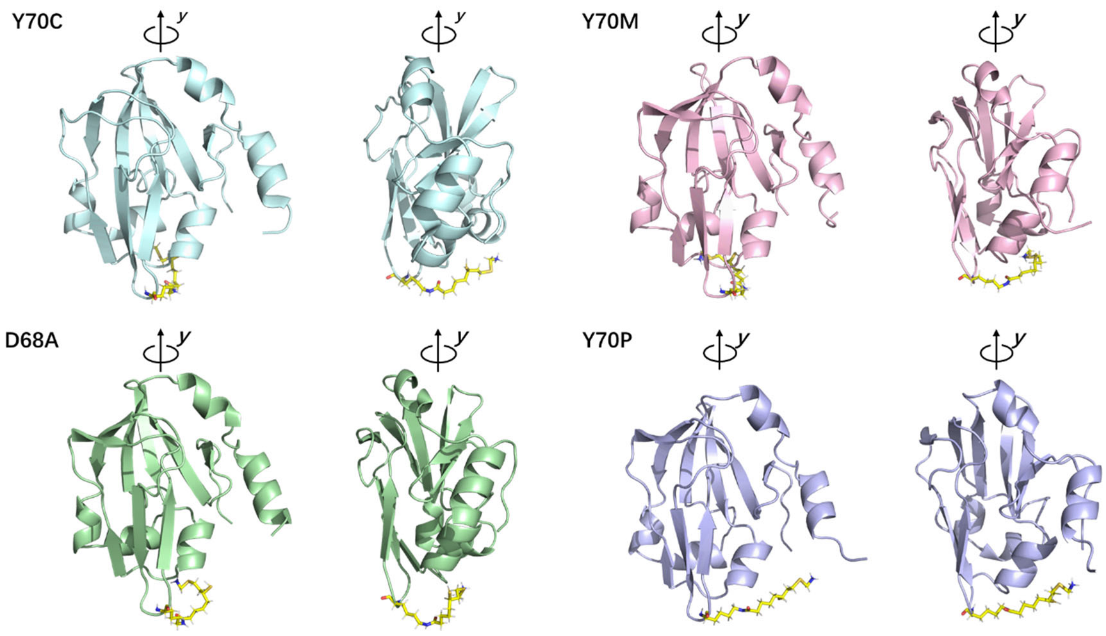

Supplementary Fig.13 The confirmation of Y70C, Y70M, D68A and Y70P at the time point highlighted in Fig.12. For Y70C and Y70M mutants, the aminomethyl lipoate arms are not locked in the hydrophobic cavity, but trapped by the α-helix in another position. By contrast, in Y70P, Y70W, Y70R, Y70F and Y70A, we have not observed such a conformational change.

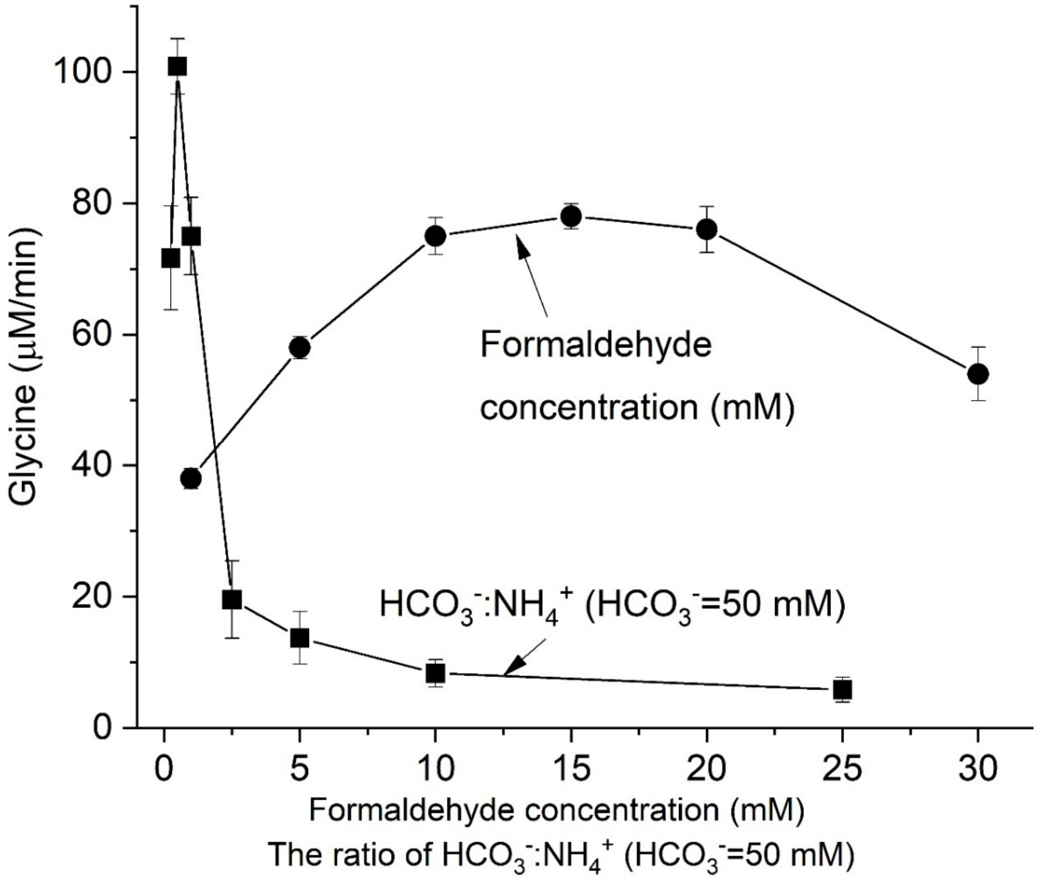

Supplementary Fig. 14 Effect of formaldehyde concentration and the C/N ratio, refers to HCO_3^-:NH_4^+ on glycine production rate. All the Data represent mean values ± SEM of 2 independent experiments.

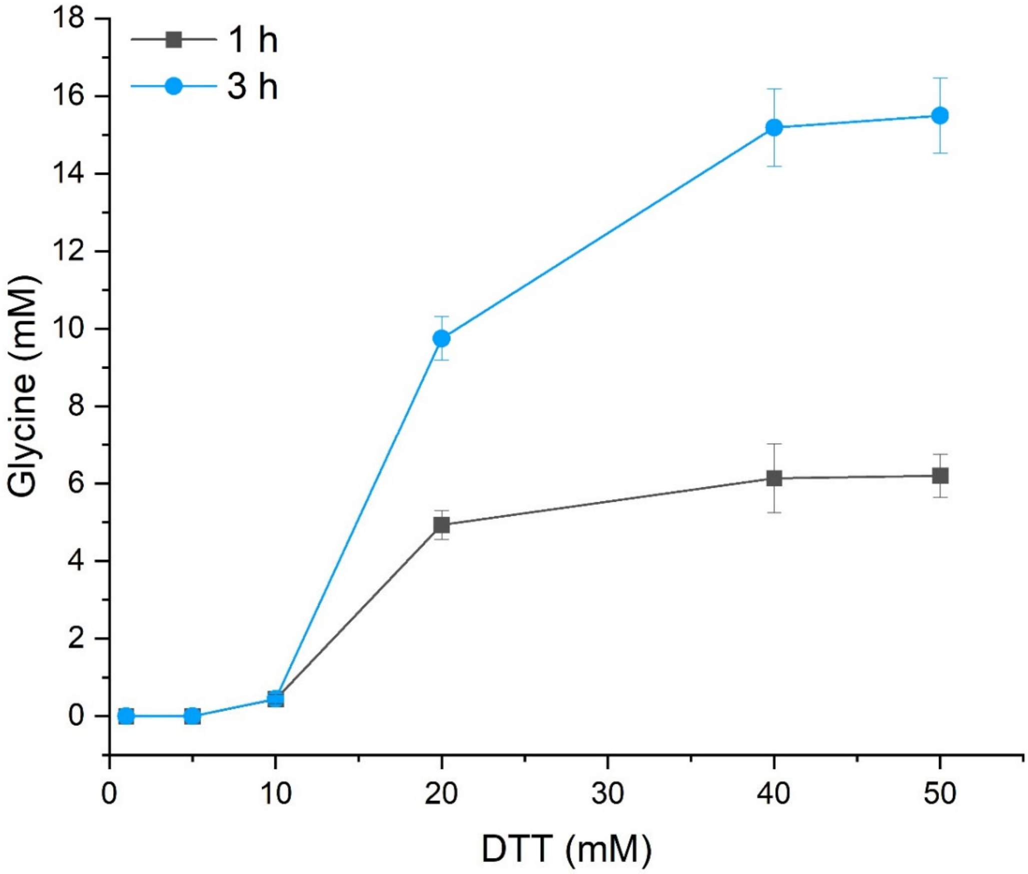

Supplementary Fig. 15 Optimization of DTT concentrations for glycine production. In this experiment, we prepared 10 mM THF as stock solution without adding DTT and the fresh THF was used immediately for the assay including the following components: H_M protein, 60 μM; P protein, 5 μM; T protein, 5 μM; HCHO, 20 mM; THF, 0.5 mM; $NaHCO_3$, 50 mM; NH_4Cl, 100 mM; PLP, 25 μM. All the Data represent mean values ± SEM of 2 independent experiments.

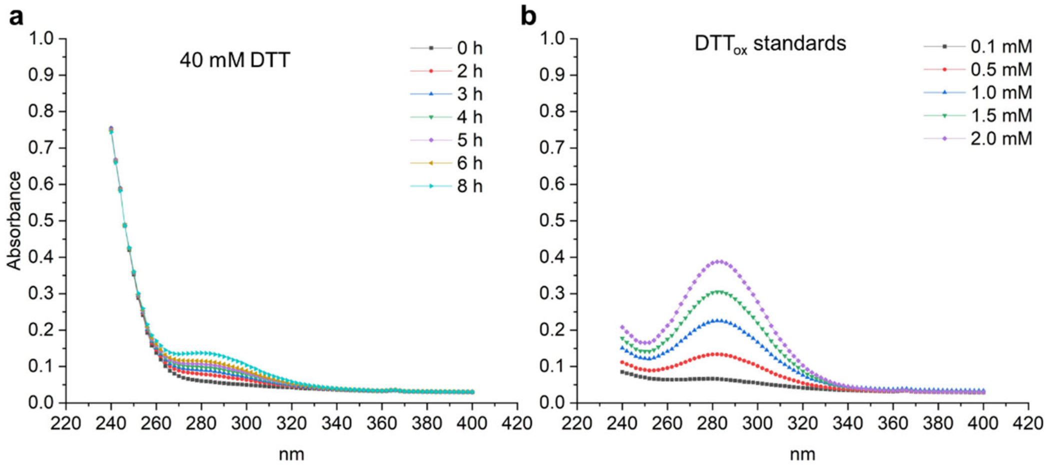

Supplementary Fig. 16 Measurement of DTT stabilities under conditions of the reaction system. **a**. DTT oxidation over time. 40 mM DTT was freshly prepared by adding DTT into 50 mM Tris-HCl (pH=7.5) buffer and the solution was placed under 37°C in the presence of air. We took samples for scanning between 240 nm and 400 nm. **b**. DTT$_{ox}$ has a specific absorbance at 280 nm.

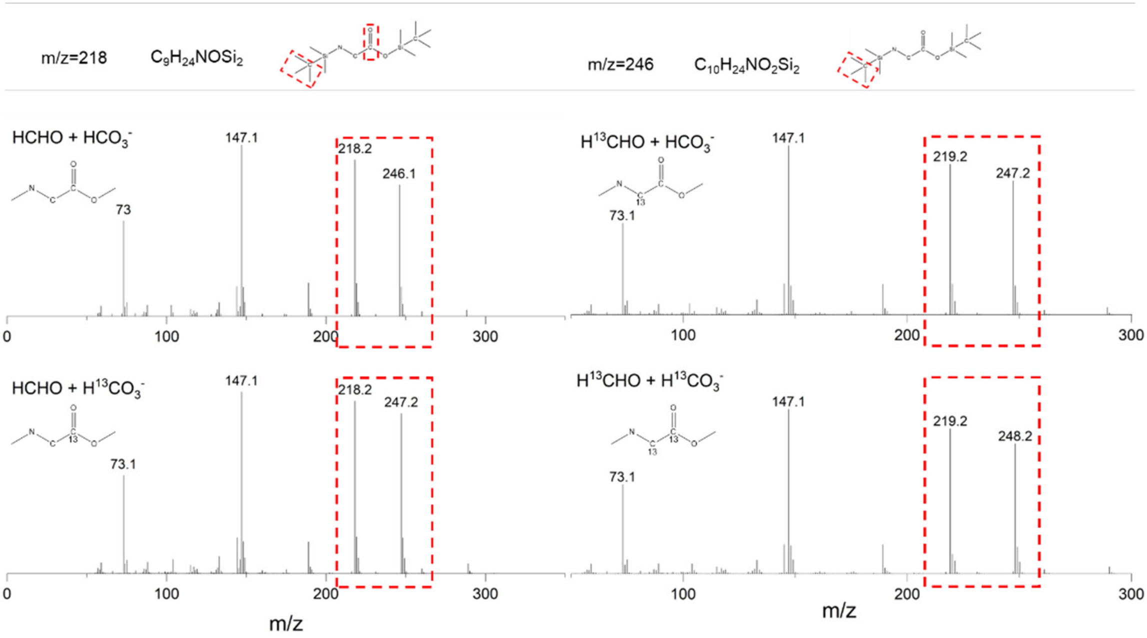

Supplementary Fig. 17 The mass spectrogram of glycine 2TBDMS (tert-Butyldimethylsilyl) derivative under different environmental conditions. We carried out $NaH^{13}CO_3$, $H^{13}CHO$, and $NaH^{13}CO_3$ + $H^{13}CHO$ labelling experiments, respectively. The carbon donor in the reaction system containing Tris-HCl (50 mM, pH 7.5), 20 mM DTT, 0.5 mM THF, 10 mM HCHO, 50 mM $NaHCO_3$, 100 mM NH_4Cl, 25 µM PLP, 5 µM P-protein, 5 µM T-protein, and 60 µM Hox, was replaced by the corresponding 10 mM $H^{13}CHO$, 50 mM $NaH^{13}CO_3$ and their combinations.

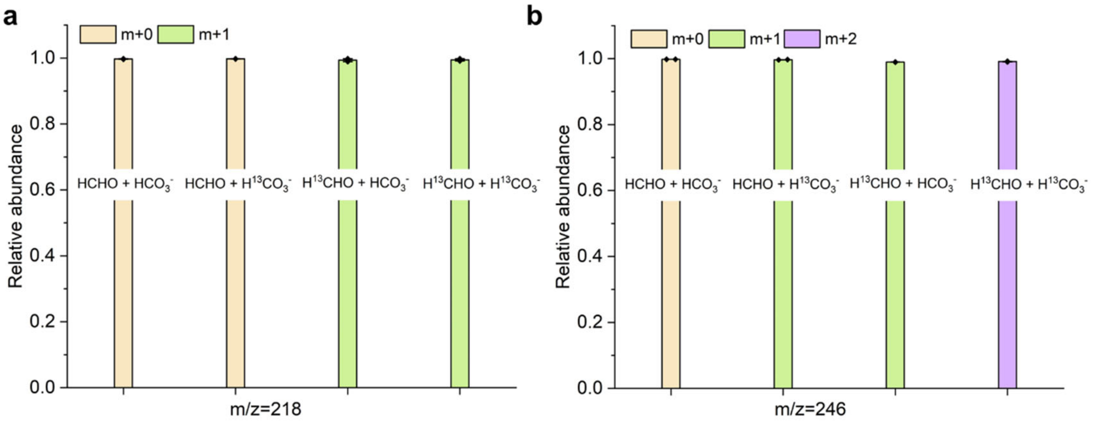

Supplementary Fig. 18 Relative abundance of glycine mass isotopomer with ^{13}C-labelling HCO_3^- or (and) HCHO as the carbon donor. **a**, m/z=218. **b**, m/z=246. Bars represent mean values ± SEM of 2 independent experiments.

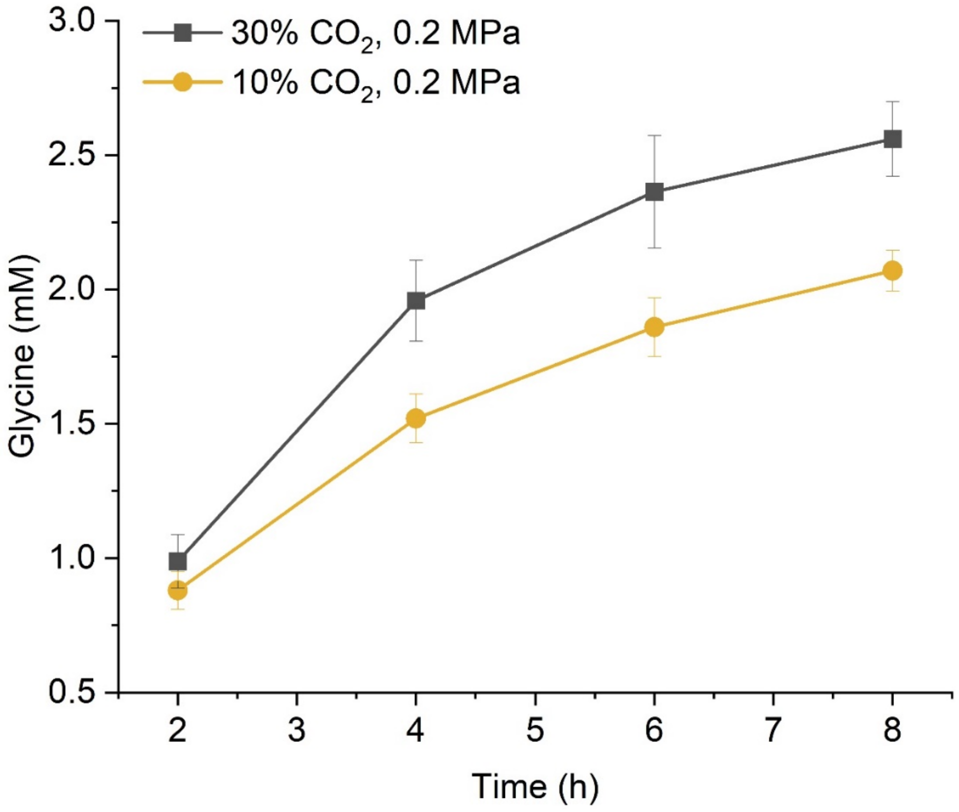

Supplementary Fig. 19 Glycine production from gaseous CO_2 (10% CO_2 with 90% air, 30% CO_2 with 70% air) under 0.5 MPa. The rGCS solution without bicarbonate was mixed in a parallel 50 ml pressure reactor (WATTCAS, China) to a final volume of 10 ml. The reaction was started by the introduction of gaseous CO_2. All the Data represent mean values ± SEM of 2 independent experiments.

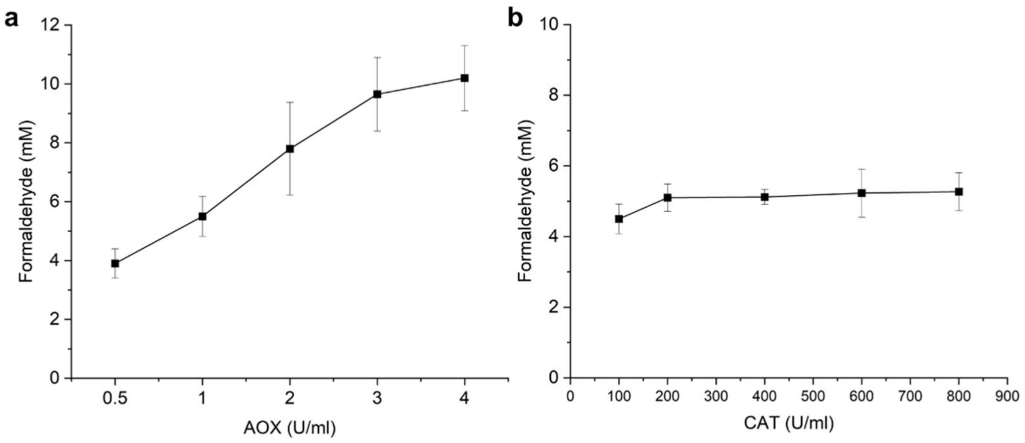

Supplementary Fig. 20 Conversion of methanol to formaldehyde using different concentrations of alcohol oxidase from *Pichia pastoris* (AOX , Sigma A2404) and catalase from bovine (Yuanye Bio-Technology, Shanghai). **a**, Optimization of the AOX (U/ml). **b**, Optimization of the CAT (U/ml). The assay containing 20 mM methanol and 40 mM MOPS buffer (pH 7.5) was performed at 37ºC. All the Data represent mean values ± SEM of 2 independent experiments.

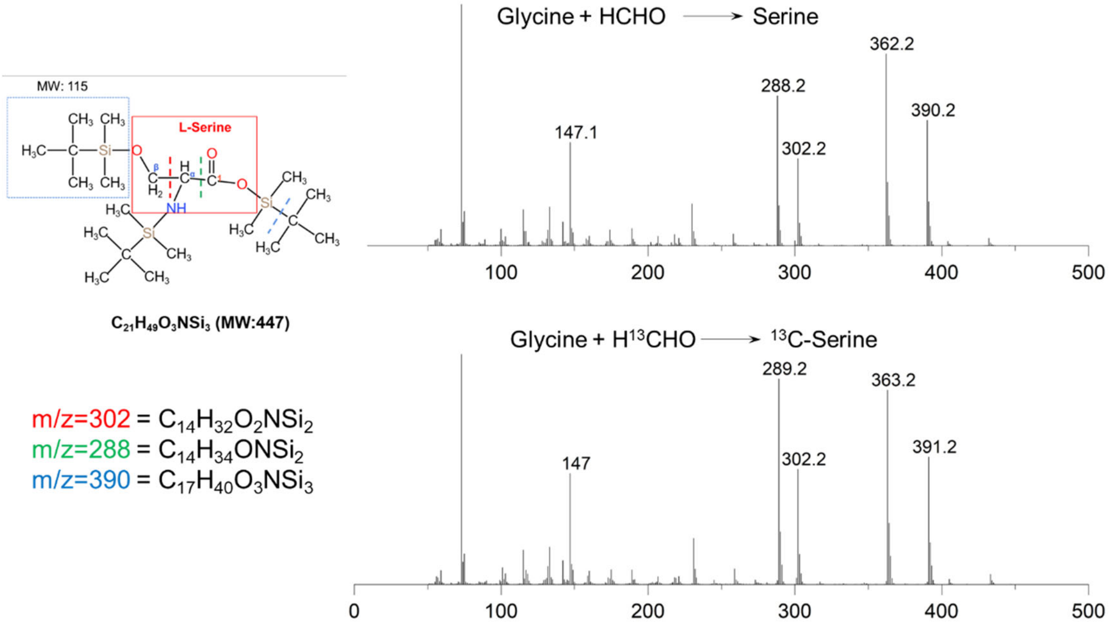

m/z=302 = $C_{14}H_{32}O_2NSi_2$
m/z=288 = $C_{14}H_{34}ONSi_2$
m/z=390 = $C_{17}H_{40}O_3NSi_3$

Supplementary Fig.21 The mass spectrogram of serine 2TBDMS derivative to confirm that ^{13}C- labeled $H^{13}CHO$ is added into the β−C of serine.

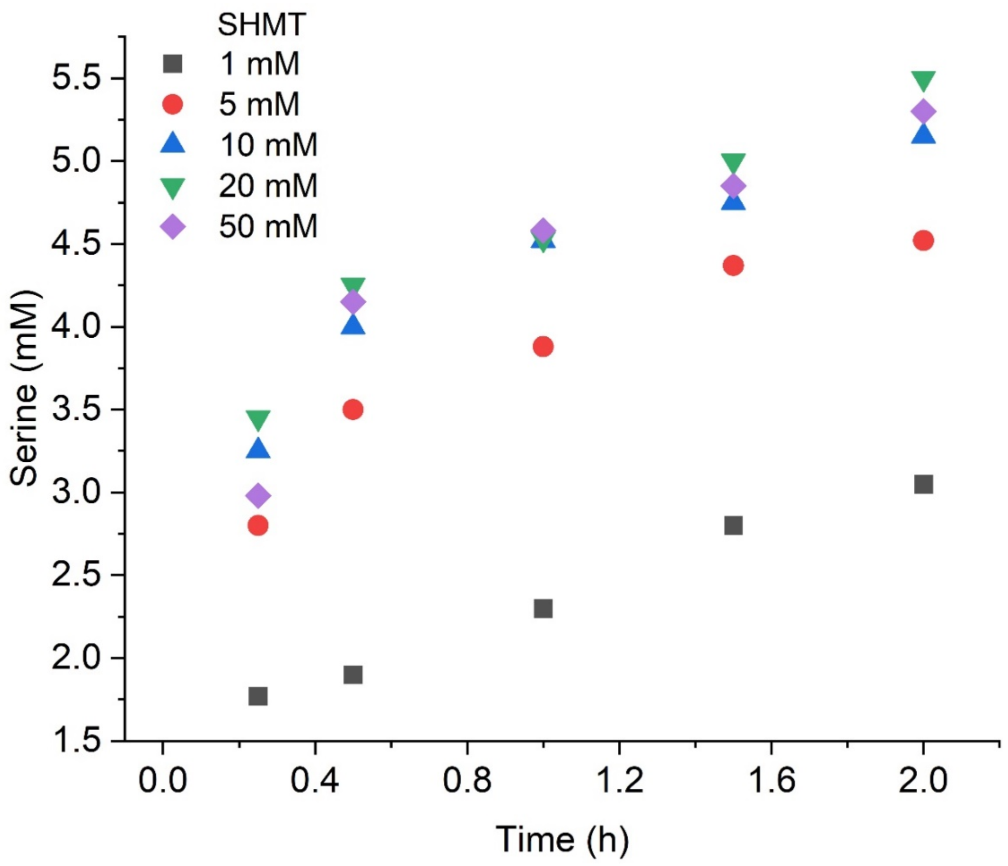

Supplementary Fig. 22 Transformation of glycine to serine using different concentrations of SHMT. The reactions were carried out in the presence of 10 mM glycine, 10 mM formaldehyde, 0.5 mM THF, pH 7.5 MOPS buffer.

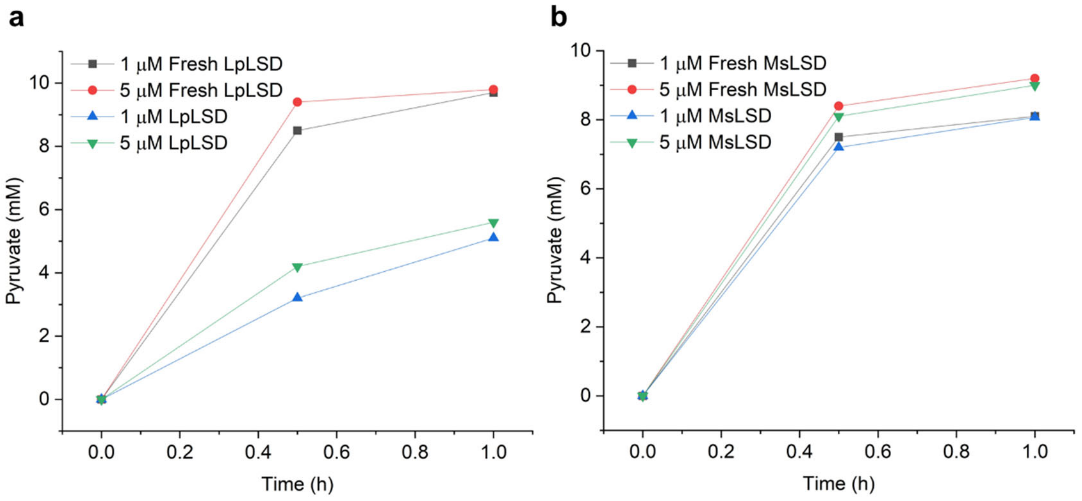

Supplementary Fig. 23 Transformation of serine to pyruvate. **a**. LpLSD was used to turn 10 mM serine into pyruvate at a pH 7.5 MOPS buffer. **b**. MsLSD was used to turn 10 mM serine into pyruvate at a pH 7.5 MOPS buffer. “Fresh” means fresh proteins purified from the recombinant *E. coli* BL21 cells and its immediate use in the enzymatic assay, while the control refers to proteins after 1 week storage in -80ºC freezer.

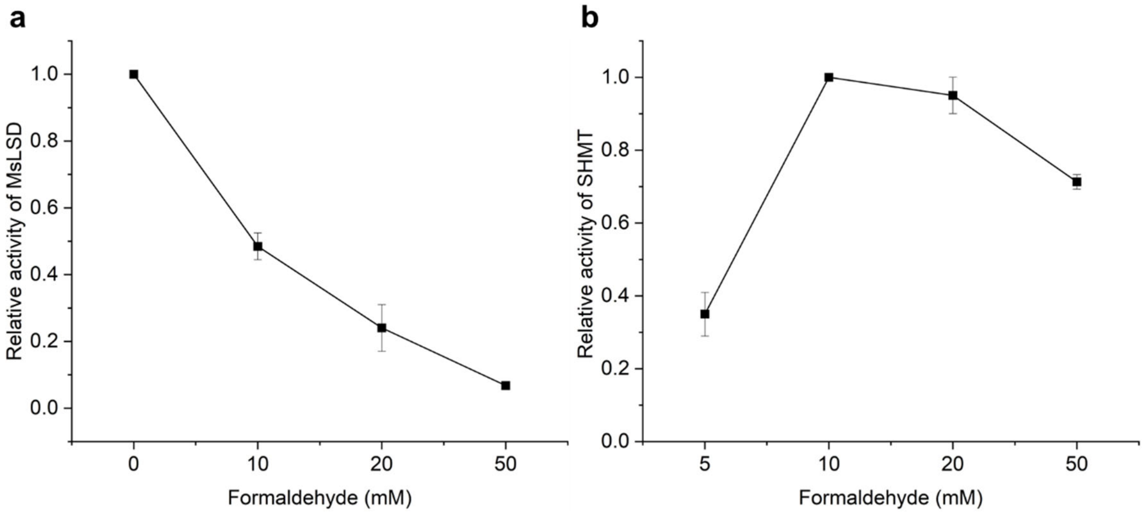

Supplementary Fig. 24 The effect of formaldehyde on the activity of MsLSD and SHMT. **a**. For testing MsLSD, 10 mM serine and 5 μM MsLSD (plus 25 μM PLP) in 40 mM MOPS buffer were incubated at 37ºC. Pyruvate was detected after 1 h reaction. It was set as 100% activity of MsLSD for the reaction without formaldehyde. **b**. For testing SHMT, the reaction containing 5-50 mM formaldehyde, 0.5 mM THF, 10 mM glycine, 20 μM SHMT was performed at 37 ºC in 40 mM MOPS buffer. The sample was taken after 1 h reaction and the activity of SHMT was set at 100% in the presence of 10 mM formaldehyde (the condensation of formaldehyde and THF can generate 5,10-CH2-THF, which is one substrate for SHMT). All the Data represent mean values ± SEM of 2 independent experiments.

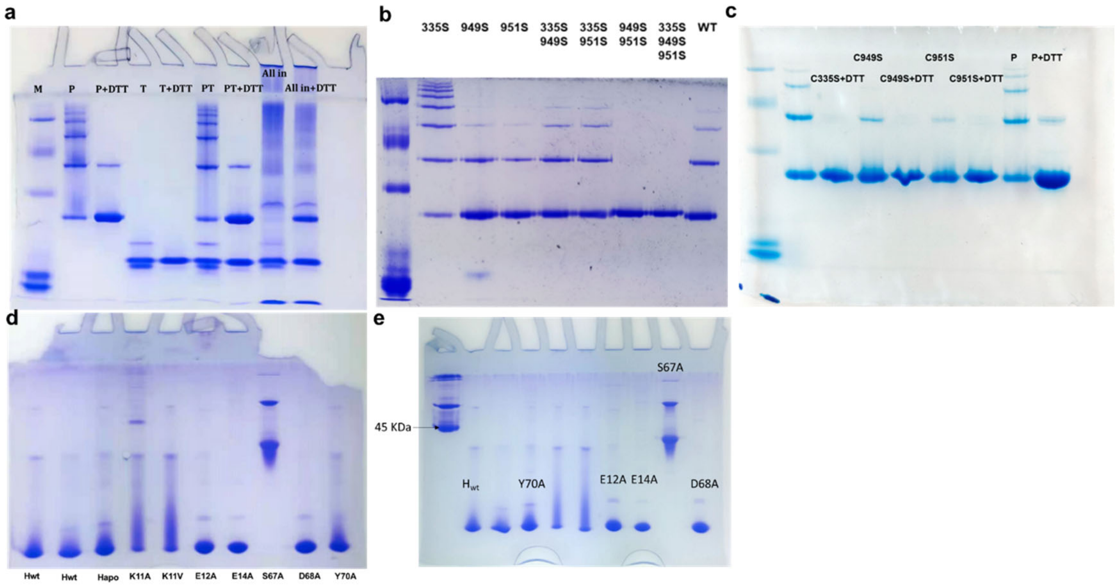

Supplementary Fig. 25 The original gels (uncropped figures) for Fig. 3f (**a**,**b**) and Fig. 4c (**d**,**e**) and supplementary Fig. 10 (**c**).

Supplementary Table 1 Strains and plasmids used in this study

	Description	Reference
***E. coli* Strains**		
DH5a	Host for cloning plasmids	WEIDI Ltd.
BL21 (DE3)	Host for protein overexpression and purification	WEIDI Ltd.
**Plasmids**		
pET28a (+)	Plasmid for protein overexpression	Novagen
pET22b (+)	Plasmid for protein overexpression	Novagen
pET28a-P	pET28a vector containing P-protein gene (NCBI No. WP_112929453.1)	This study
pET28a-P-C335S	pET28a vector containing P-protein gene with point mutation of C335S	This study
pET28a-P-C949S	pET28a vector containing P-protein gene with point mutation of C949S	This study
pET28a-P-C951S	pET28a vector containing P-protein gene with point mutation of C951S	This study
pET28a-P-C949S, C951S	pET28a vector containing P-protein gene with point mutations of C949S and C951S	This study
pET28a-T	pET28a vector containing T-protein gene (NCBI No. WP_099356926.1)	Xu et al. 2022
pET28a-L	pET28a vector containing L-protein gene (NCBI No. WP_110826218.1)	Xu et al. 2022
pET28a-H	pET28a vector containing H-protein gene (NCBI No. WP_001295377.1)	Zhang et al. 2020
pET28a-H-E12A	pET28a-H containing H-protein gene with point mutation of E12A	Zhang et al. 2020
pET28a-H-E14A	pET28a-H containing H-protein gene with point mutation of E14A	Zhang et al. 2020
pET28a-H-S67A	pET28a-H containing H-protein gene with point mutation of S67A	Zhang et al. 2020
pET28a-H-D68A	pET28a-H containing H-protein gene with point mutation of D68A	Zhang et al. 2020
pET28a-H-Y70A	pET28a-H containing H-protein gene with point mutation of Y70A	Zhang et al. 2020
pET28a-H-Y70M	pET28a-H containing H-protein gene with Y70 saturated mutagenesis library	This study
pET22b-SHMT	pET28a vector containing SHMT gene ((NCBI No. WP_226145048.1)	This study
pET28a-LpLSD	pET28a vector containing LSD gene from *Legionella pneumophila* (NCBI No. WP_010946527.1)	This study
pET28a-MsLSD	pET28a vector containing LSD gene from *Mycolicibacterium smegmatis* (NCBI No. WP_011729138.1)	This study

Supplementary Table 2. Primers used in this study

Primer	Sequence
pET28a-P-F	5'-catgccatgggcacacagacgttaagccagcttgaaaac-3'
pET28a-P-R	5'-ctgctcctgcgtaccgattagcgaataccagctcgagcgg-3'
pET28a-T-F	5'-catgccatggcacaacagactcctttgtacgaacaa-3'
pET28a-T-R	5'-ccgctcgagcgcgacggctttgccgttacgcacaaaaac-3'
pET28a-H-F	5'-catgccatgggcagcaacgtaccagcagaactgaaatac-3'
pET28a-H-R	5'-ccgctcgagctcgtcttctaacaatgcttcgtatgc-3'
pET28a-L-F	5'-gggaattccatatgatgagtactgaaatcaaaactcaggtcg-3'
pET28a-L-R	5'-ccgctcgagttacttcttcttcgctttcgggttc-3'
pET28a-C335S-F	5'-gcgaactccaacatttctacttcccaggtactg-3'
pET28a-C335S-R	5'-aatgttggagttcgctttctcacg-3'
pET28a-C949S-F	5'-tcctgcgtaccgattagcgaatac-3'
pET28a-C949S-R	5'-ctaatcggtacgcaggaagagaacaggttacggtc-3'
pET28a-C951S-R	5'-gtattcgctaatcggtacagaggagcagaacaggttacg-3'
pET28a-C949S, C951S-F	5'-gtaccgattagcgaataccagctcg-3'
pET28a-C949S, C951S-R	5'-gtattcgctaatcggtacagaggaagagaacaggttacggtcgcc-3'
pET22b-SHMT-F	5'-cccaagcttttagtggtggtggtggtggtgtgcgtaaaccgggtaacg-3'
pET22b-SHMT-R	5'-ggaattccatatgatgttaaagcgtgaaatgaacattgc-3'
pET22b-LSD-F	5'-ggaattccatatgatgcaccaccaccaccaccacaacattagtgtttttgatttattttctataggaattg-3'
pET22b-LSD-R	5'-gcaagcttctaacactcaggcaagttaac-3'
pET28a-Y70M-F	5'-aaagcggcgtcagacattnnkgcgccagtaagcggtgaaat-3'
pET28a-Y70M-R	5'-aatgtctgacgccgcttttactga-3'

Supplementary Table 3. Reduction potential of some biological redox couples

Reduction			Standard reduction potential (V)
β-ME_{ox} + 2 H^+ + 2 e^-	⟶	β-ME	-0.26
Lipoic acid + 2 H^+ + 2 e^-	⟶	dihydrolipoic acid	-0.29
$TCEP_{ox}$ + 2 H^+ + 2 e^-	⟶	TCEP	-0.32
DTT_{ox} + 2 H^+ + 2 e^-	⟶	DTT	-0.33

Supplementary Table 4. Thermadynamic characterizations of glycine production pathways[1]

Reactions			$\Delta_r G_m$'(kJ/mol)
Formate + THF + ATP	⟶	10-formyl-THF + ADP	-4.8
10-formyl-THF	⟶	5,10-methenyl-THF	5.0
5,10-methenyl-THF + NADPH	⟶	5,10-methylene-THF + $NADP^+$	-9.9
5,10-methylene-THF + NH4 + CO2 + NADH	⟶	Glycine + THF + NAD^+	-1.2
Formate + ATP + CoA	⟶	Formyl-CoA + ADP	-28.2
Formyl-CoA + NADH	⟶	Formaldehyde + NAD^+	27.5
Formaldehyde + THF	⟶	5,10-methylene-THF + H_2O	-21.1
Formate + ATP	⟶	Formyl-P	-18.0
Formyl-P + CoA	⟶	Formyl-CoA	-4.2
Formate + NADH	⟶	Formaldehyde + NAD^+	54.8
Methanol + NAD^+	⟶	Formaldehyde + NADH	45.1
Methanol + O_2	⟶	Formaldehyde + H_2O_2	-100.6

[1] thermodynamic analysis of $\Delta_r G_m$' for individual steps of the pathways under physiological conditions (pH 7.5, ionic strength I=0.20, sustrates = 1 mM, ATP = 2 mM, ADP = 2 mM, NADH = 2 mM, NAD^+ = 2 mM, CoA = 0.5 mM, THF = 0.5 mM).

Supplementary Table 5. The synthesized LSD enzymes in this study

MsLSD (*Mycolicibacterium smegmatis*)
MDLVTLDDISGAAARIAADIVRTPLLAADWGDPRCPLWLKAETLQPIGAFKIRGAFNALG<br>RLDTHTRARGVVAYSSGNHAQAVAYAAAAYGVPAHIVMPEETPAVKVEATRRRGAHVVL<br>CGAGERERTAAELVEKTGAVLIPPFDHPDIIAGQGTIGIEIAEDLPELATVLIPVSGGGLASGI<br>GTAIRALRPKAKIFAVEPELAADTAESLALGSIVEWPVAKRNRTIADGLRSTPSELTFAHLR<br>QVIDDVITVSEDEIRSAVRELALRARLVAEPSGAVSLAGYRKAALPDGSAVAIVSGGNIEPA<br>QLAAILAGG
LpLSD (*Legionella pneumophila*)
MNISVFDLFSIGIGPSSSHTVGPMLAANAFLQLLEQKNLFDKTQRVKVELYGSLALTGKGH<br>GTDKAILNGLENKAPETVDPASMIPRMHEILDSNLLNLAGKKEIPFHEATDFLFLQKELLPK<br>HSNGMRFSAFDGNANLLIEQVYYSIGGGFITTEEDFDKSTTDTNPPPYPFATATELLKLCKK<br>HHLTIAELMLVNEKTWRSSAEIHKGILDIAKVMDDCINNGCKHDGVLPGGLNLKRRAPDL<br>YRKLIEQKGVKSVFEQSDIMNHLNLYAMAVNEENAAGGRIVTAPTNGAAGIIPAVLKYCQ<br>QAHDRMSNEDIYTYFLTAAAIGILYKKGASISGAEVGCQGEVGVASSMAAAGLTAVLGGTI<br>EQVENAAEIAMEHHLGMTCDPVLGLVQIPCIERNAMGAVKAVNATRMALIGDGQHQISLD<br>KVIKTMKQTGMDMQSIYKETSMGGLAVNLPEC
